# The importance of enjoyment, sensory properties and perceived cooking abilities in legume and pulse consumption: a questionnaire study

**DOI:** 10.1017/S1368980024001058

**Published:** 2024-05-07

**Authors:** Katherine Marie Appleton

**Affiliations:** Research Centre for Behaviour Change, Department of Psychology, Bournemouth University, Poole, BH12 5BB, UK

**Keywords:** Healthy diets, Sustainable diets, Barriers, Facilitators, Cross sectional

## Abstract

**Objective:**

Legume and pulse consumption is currently recommended for health and sustainability purposes, but barriers to consumption can include low enjoyment and poor sensory properties. This work aimed to investigate the relative importance of a number of barriers and facilitators towards legume, including pulse, consumption with a specific focus on enjoyment, sensory properties and a possible role for perceived cooking abilities in these relationships.

**Design:**

A cross-sectional questionnaire study assessed legume and pulse consumption, agreement and disagreement with statements relating to enjoyment, sensory properties, cooking abilities, practical aspects, healthiness, upbringing, social influences and quality issues, and four demographic characteristics. Complete responses were gained from 633 respondents with a mix of genders, ages, usual cooking responsibilities and usual eating habits.

**Setting:**

UK, March 2021 – September 2022.

**Participants:**

General UK adult population.

**Results:**

Using multiple regression analyses, enjoyment and cooking abilities were found to be important for both legume and pulse consumption (smallest beta = 0·165, *P* < 0·01), and the sensory properties of these foods were also important for the consumption of pulses (beta = 0·099, *P* = 0·04). Perceived cooking abilities also reduced the importance of enjoyment and sensory properties for consumption, mitigated effects due to upbringing and practical aspects and increased the value of perceived health benefits (smallest beta = 0·094, *P* = 0·04).

**Conclusions:**

These findings demonstrate a clear role for enjoyment, sensory properties and perceived cooking abilities in legume and pulse consumption and suggest benefits for increasing cooking abilities for improved legume and pulse consumption, as result of both direct and indirect effects.

Legumes are the edible parts of plants from the botanical family *Leguminosae* or *Fabaceae,* including their fruits and seeds. Leguminous crops can be used for food, feed and sowing, where crops for food are either harvested green, to include green beans, fresh peas, peanuts and soybeans, or are harvested for dry grain only. Those harvested only for dry grain are referred to as pulses and include dry beans, dry peas, chickpeas and lentils^(1)^.

Legumes, including pulses, are high in protein, and complex carbohydrates, including resistant starch, have soluble and insoluble fibres and are low in saturated fat^(2–6)^. They are also high in many micronutrients, including B-vitamins, potassium, Mg, Fe, Cu, Zn and phosphorus, and contain many other bioactive compounds, such as phytochemicals, polyphenols and flavonoids^(2–6)^. Some reduced absorption and utilisation of micronutrients can occur as a result of these co-existing non-nutrients or anti-nutrients^(2–5)^, but many of these bioactive compounds also provide health benefits via anti-oxidant, anti-inflammatory and anti-cancer actions^(2–5)^, and processing and preparatory techniques can reduce their negative effects^(2–5)^. The high nutrient density of legumes is reflected in the improved nutrient profiles of high *v*. low consumers of legumes^(7,8)^.

The nutritional components confer considerable health benefits, and as such, the consumption of legumes is associated with improved health. Legume consumption has been associated with improved cardiovascular health and hypertension^(3,5,6,9–12)^, blood sugar and blood lipid profiles^(3,5,6,9,12)^, body weight and body weight maintenance^(3,5,6,9,11,12)^ and may have important anti-oxidant, anti-inflammatory and anti-cancer actions, as above^(2,3,5,9,13)^. Legume consumption may also have health benefits as a result of the displacement of less healthy foods, such as red meat, processed meats and less complex carbohydrates, within the diet^(4,10,13)^.

Legume consumption also incurs a low environmental footprint. Greenhouse gas emissions are reported to be around 0·8–3·5 kgCO^2^/kg for legumes, pulses and nuts, compared with 5–8 kgCO^2^/kg for meat from poultry and pigs and considerably higher values for other meats^(14,15)^. Freshwater withdrawals (scarcity-weighted) are comparable for pulses, eggs and poultry, at approximately half of that required for grains and beef, although freshwater requirements for peas and peanuts are almost twice that for pulses^(15)^. Conversely, land use is comparable for pulses, pig meat, cheese and eggs, while land use for peas and peanuts is lower^(15)^. Legumes are also well adapted for growth in adverse environmental conditions, have high disease resistance and many legumes house nitrogen-fixing bacteria in root nodules and so can increase the fertility of the soil^(2,3,16)^. This increased fertility can reduce the need for chemical fertilisers and increase the productivity of following crops, providing an important role for legumes in crop rotation and further increasing the environmental benefits^(2,3,16)^.

For health and sustainability reasons, the Eat Lancet Planetary Health diet currently recommends consumption of at least 75 g/d legumes^(17,18)^. Consumption in the Western World, however, is low. Consumption of legumes and nuts in the UK was most recently reported as 31 g/d^(19)^. In Europe, consumption ranges between 9 g/d–26 g/d, resulting in an average consumption of 15 g/d^(19)^, and similar low levels of consumption are also reported in the US and Canada^(4,11,12)^.

Reasons for this low consumption include a lack of enjoyment and low liking for the sensory properties of legumes^(20–24)^, low familiarity with legumes^(20,22,24,25)^, low knowledge of their health benefits^(20,24,26)^, perceptions that legumes are time consuming and effortful to prepare and cook^(4,21,23–28)^, a lack of relevant cooking knowledge, skills and experience^(20–24,26,27,29)^, perceptions that legumes can cause gastrointestinal effects such as flatulence and bloating^(2,4,20,25,26)^ and perceptions that legumes are unsuitable for my identity or lifestyle^(4,20–22,26,27)^.

Of these, lack of enjoyment and disliking of sensory properties are often reported as key reasons for low consumption of healthy foods, particularly vegetables^(30–34)^. Recent work, furthermore, suggests some recognition that cooking abilities may provide solutions to these barriers. Simple cooking suggestions, such as incorporating pulses into existing dishes such as soups and stews, may access existing acceptance and liking^(21–24,27,33,35)^, and the use of recipes for tasty meals can offer new opportunities^(21,23,24,26,27,35)^. Limited evidence of impact, however, is currently available, and no study, as far as we are aware, has investigated the value of cooking abilities specifically for addressing enjoyment or sensory concerns.

This work aimed to investigate the relative importance of a number of barriers and facilitators towards legume, and pulse, consumption, with a specific focus on enjoyment, sensory properties and a possible role for cooking abilities in these relationships.

## Methods

The study used a cross-sectional questionnaire design, where consumption of legumes, including pulses, a number of attitudes towards legumes and a number of demographic characteristics were assessed at a single time point.

### Questionnaire

Legume and pulse consumption was assessed using a FFQ requesting frequency of consumption of: ‘*baked beans*’; ‘*kidney, cannellini or borlotti beans*’; ‘*black, pinto or butter beans*’; ‘*broad or fava beans*’; ‘*other beans*’; ‘*lentils*’; ‘*chickpeas*’; ‘*peas*’ and ‘*peanuts*’. All these foods were considered to be legumes. All beans, chickpeas and lentils, but not peas and peanuts, were also considered to be pulses. Response options were: ‘*every day*’, ‘*3–5 times a week*’, ‘*1–2 times a week*’, ‘*1–2 times a fortnight*’, ‘*1–2 times a month*’, ‘*less than once a month*’ and ‘*never*’, as utilised in a validated FFQ^(36)^. Response options were scored: 7, 4, 1·5, 0·5, 0·25, 0·05 and 0, respectively, to provide a frequency relative to once per week. Consumption of a number of additional foods that also contribute protein to the diet^(37)^ was also requested to increase the face validity of the questionnaire. These foods were dairy foods: milk, yoghurt, custards and blancmanges, soft cheeses (e.g. cream cheese, Dairylea and camembert) and hard cheeses (e.g. cheddar, stilton and emmental); eggs; nuts (other than peanuts) and protein substitutes (e.g. Quorn).

Barriers and facilitators towards legume and pulse consumption were assessed using a number of attitudinal statements, to which participants were asked to agree or disagree. A number of statements varied per barrier/facilitator depending on the complexity of the factor and were a mix of positive and negative statements. The factors assessed were enjoyment (two items); sensory properties (eight items); perceived cooking abilities (ten items); perceived practical aspects (six items); perceived healthiness (two items); upbringing (two items); social influences (four items) and quality issues (six items). These factors were based largely on our recent work^(24)^, where barriers and facilitators towards consuming pulses were explored in interviews with thirty-three UK adults before and after receiving pulse-based cooking suggestions and recipes. The factor relating to quality issues did not stem from this work and was taken instead from literature on the consumption of animal-based protein-rich foods^(38)^. This factor was included as a control or distractor, i.e. we did not expect any associations between legume or pulse consumption and this factor. Statements for each factor are given in the online supplementary material, Supplemental Table SM1. All statements were responded to on a seven-point scale labelled ‘*strongly agree’, ‘moderately agree’, ‘slightly agree’, ‘neither agree nor disagree’, ‘slightly disagree’, ‘moderately disagree’ and ‘strongly disagree*’. Responses were scored +3 to –3, respectively, such that higher scores denoted greater agreement. Composite scores for each factor were created by reverse scoring negative statements, adding the responses to all relevant items and dividing by the number of these. Thus, composite scores for all factors contributed between a possible maximum of +3 and a possible minimum of –3 for all analyses.

Four demographic characteristics were also queried: gender (male, female, non-binary/other); age (years); usual cooking responsibilities (usually cook for myself, usually cook for others, usually cooked for) and usual eating scenario (usually eat by myself, usually eat with others). These demographic characteristics have previously been associated with legume or pulse consumption^(7,8,20,23,25,27–29)^. Two additional questions also asked if participants ‘*don’t eat pulses for medical reasons*’ or ‘*have been told not to eat pulses by doctors*’. Participants agreeing to these items were removed from the dataset, as their intakes may not have reflected their attitudes.

### Questionnaire administration

The complete questionnaire was made available to staff and students of Bournemouth University, UK and their contacts, to a Bournemouth University participant pool composed of members of the UK public and to participants of ongoing unrelated studies in the Eating Behaviours Laboratory of Bournemouth University, UK, from March 2021 to September 2022. Recruitment focussed on young adults, as a population group who may be more amenable to dietary change, and where benefits may accrue over the long term^(39)^. Volunteers of all ages, however, were welcomed. Respondents were required to be aged 18 years and over, able to read and understand English and able to provide informed consent. All questionnaires were completed online. Consenting procedures were also completed online in advance of questionnaire completion.

### Analyses

First questionnaires were screened for completeness and questionnaires with missing data or with responses resulting in unlikely high dietary intakes were removed. Questionnaires were also removed from any respondent who reported being younger than 18 years or reported not consuming legumes/pulses for medical reasons. Second, checks for parametric data were undertaken, composite scores for all factors were created and the sample was described. Third, a series of regression models were run for both legume consumption and pulse consumption. Details of the variables included in each model are given in Table 1. Models 1 and 2 investigate associations between legume/pulse consumption, the four demographic characteristics and the attitudes related to practical aspects, healthiness, upbringing, social influences and quality issues. Model 3 demonstrates the added value of enjoyment in legume/pulse consumption. Model 4 demonstrates the impact of perceived cooking abilities on these effects. Model 5 demonstrates the added value of their sensory properties in legume/pulse consumption. Model 6 demonstrates the impact of cooking abilities on these effects. Prior to all analyses, checks for multi-co-linearity revealed no concerns. Analyses were conducted in IBM SPSS, version 28. Significance was set at *P* < 0·05.

**Table 1:** The variables included in each regression model

Model	Variables included		
1	Demographic variables: gender, age, usual cooking responsibilities, usual eating scenario		
2	Model 1, plus Attitudes: practical aspects, healthiness, upbringing, social influences, quality issues		
3	Model 2, plus Attitudes: enjoyment	5	Model 2, plus Attitudes: sensory properties
4	Model 3, plus Attitudes: cooking abilities	6	Model 5, plus Attitudes: cooking abilities

## Results

### Participant sample

Complete questionnaires from 647 respondents were returned. Of these, eleven individuals reported not consuming pulses for medical reasons and three individuals reported consuming legumes or pulses more than three times per day on average; these questionnaires were removed from the dataset. A final sample of 633 respondents was included in all analyses. Descriptive statistics detailing the demographic characteristics for the final sample and frequency of legume and pulse consumption are given in Table 2.

**Table 2:** Descriptive statistics (*n* or mean (sd) and range (minimum to maximum)) for the final sample (*n* 633)

Variable	*n* or mean (sd)	Range (min–max)
Gender (*n* (%))	Males: 114 (18 %); female: 516 (81·5 %); non-binary gender: 3 (0·5 %).	
Age (years)	31·8 (14·4) years	18–75 years
Age (*n* (%))	18–20 years: 235 (37 %); 21–30 years: 133 (21 %); 31–40 years: 78 (12 %); 41–50 years: 94 (15 %); 51–60 years: 66 (11 %); 61–75 years: 27 (4 %).	
Usual cooking responsibilities (*n* (%))	Usually cooked for themselves: 274 (43 %), usually cooked for others: 274 (43 %); usually cooked for: 85 (14 %).	
Usual eating situation (*n* (%))	Usually ate by themselves: 206 (33 %); usually ate with others: 427 (67 %).	
Legume consumption (times/week)	4·1 (3·5) times/week	0–21 times/week
Legume consumption (*n* (%))	Less than once/week: 103 (16 %); Once or twice/week: 160 (25 %); 3–5 times/week: 199 (32 %); Every day or almost every day: 70 (11 %); More than once a day: 101 (16 %).	
Pulse consumption (times/week)	2·3 (2·5) times/week	0–18·5 times/week
Pulse consumption (*n* (%))	Less than once/week: 252 (40 %); once or twice/week: 195 (30 %); 3–5 times/week: 125 (20 %); every day or almost every day: 35 (6 %); more than once a day: 26 (4 %).	

### Barriers and facilitators

Descriptive statistics for the responses on all attitudinal scales are given in Table 3. All factors were independent of each other (*r* = 0·005, *P* = –0·90 to *r* = 0·553, *P* < 0·01), with the exception of the factors: Enjoyment and sensory properties (*r* = 0·797, *P* < 0·01). All factors were significantly positively associated with legume consumption when tested individually (*r* = 0·182, *P* < 0·01 to *r* = 0·312, *P* < 0·01), with the exception of the factor: Quality issues (*r* = –0·027, *P* = 0·50). All factors were significantly positively associated with pulse consumption when tested individually (*r* = 0·202, *P* < 0·01 to *r* = 0·340, *P* < 0·01), with the exception of the factor: Quality issues (*r* = –0·028, *P* = 0·48).

**Table 3:** Descriptive statistics (Cronbach’s alpha, mean (sd) and range (minimum to maximum) for all barriers and facilitators (*n* 633)

Barrier/Facilitator	Cronbach’s *α*	Mean	sd	Range (min–max)
Enjoyment	0·914	1·0	1·6	−3·0 to 3·0
Sensory properties	0·801	0·7	1·1	−2·75 to 2·38
Cooking abilities	0·771	0·1	1·0	−2·4 to 2·8
Practical aspects	0·729	1·0	1·0	−1·5 to 3·0
Healthiness	0·763	2·0	0·9	−3·0 to 3·0
Upbringing	0·762	−0·2	1·8	−3·0 to 3·0
Social influences	0·653	1·1	1·2	−2·0 to 3·0
Quality issues	0·688	0·0	0·9	−3·0 to 2·83

### Legume consumption

In model 1, legume consumption was not significantly predicted by the demographic characteristics alone (R^2^ = 0·01, adj. R^2^ = 0·01, F(4, 632) = 0·69, *P* = 0·60). Results of all subsequent regression models are given in Table 4. Model 2 demonstrates positive associations between legume consumption and perceptions that this consumption is healthy and that participants have always eaten/been brought up eating legumes. Model 3 demonstrates positive associations between legume consumption and enjoyment. The differences found between models 2 and 3 also suggest a role for enjoyment in the earlier associations with healthiness and upbringing. Model 4 demonstrates an additional positive role for perceived cooking abilities in legume consumption. Comparison between models 3 and 4 also demonstrates a reduced role for enjoyment and an increased role for perceptions of healthiness when perceived cooking abilities are considered. Model 5 demonstrates a very limited role for sensory properties in either legume consumption or the earlier associations between legume consumption and perceptions of healthiness and upbringing. Model 6 also demonstrates no role for sensory properties in legume consumption, but again demonstrates the positive association between legume consumption and perceived cooking abilities. Comparisons between models 5 and 6 suggest a reduced role for upbringing and an increased role for perceptions of healthiness in legume consumption when perceived cooking abilities are also considered.

**Table 4:** Results of all regression analyses investigating legume consumption (*n* 633)

	2 – demographic variables, plus some attitudinal factors		3 – demographic variables, some attitudinal factors plus enjoyment		4 – demographic variables, some attitudinal factors, enjoyment and cooking abilities		5 – demographic variables, some attitudinal factors, plus sensory properties		6 – demographic variables, some attitudinal factors, sensory properties and cooking abilities	
	R^2^ = 0·09, adj. R^2^ = 0·07, F(9, 632) = 6·39, *P* < 0·01		R^2^ = 0·11, adj. R^2^ = 0·10, F(10, 632) = 7·71, *P* < 0·01		R^2^ = 0·13, adj. R^2^ = 0·11, F(11, 632) = 8·36, *P* < 0·01		R^2^ = 0·09, adj. R^2^ = 0·07, F(10, 632) = 6·00, *P* < 0·01		R^2^ = 0·11, adj. R^2^ = 0·10, F(11, 632) = 7·25, *P* < 0·01	
Regression Model	Beta	*P*	Beta	*P*	Beta	*P*	Beta	*P*	Beta	*P*
Gender	0·001	0·98	–0·007	0·85	–0·008	0·84	0·001	0·99	–0·002	0·96
Age	0·042	0·32	0·022	0·60	0·025	0·55	0·035	0·40	0·038	0·36
Eat alone/with others	–0·048	0·34	–0·054	0·27	–0·053	0·27	–0·048	0·33	–0·048	0·32
Cook for myself/for others/do not cook	0·058	0·24	0·061	0·21	0·077	0·11	0·059	0·23	0·078	0·11
Practical aspects	0·082	0·09	0·030	0·55	–0·044	0·41	0·064	0·20	–0·023	0·66
Healthiness	**0·140**	**< 0·01**	0·071	0·13	**0·101**	**0·03**	**0·128**	**< 0·01**	**0·154**	**< 0·01**
Upbringing	**0·110**	**0·02**	0·062	0·17	0·038	0·40	**0·095**	**0·04**	0·065	0·16
Social influences	0·064	0·19	0·028	0·56	0·010	0·84	0·046	0·36	0·027	0·58
Quality issues	–0·025	0·55	–0·031	0·45	–0·029	0·46	–0·022	0·60	–0·023	0·56
Enjoyment			**0·221**	**< 0·01**	**0·176**	**< 0·01**				
Sensory properties							0·075	0·13	0·025	0·62
Cooking abilities					**0·178**	**< 0·01**			**0·210**	**< 0·01**

Significant predictors (*P* < 0·05) are given in bold.

### Pulse consumption

In model 1, pulse consumption was not significantly predicted by the demographic variables alone (R^2^ = 0·01, adj. R^2^ = 0·01, F(4, 632) = 1·91, *P* = 0·11). Results of all subsequent regression models are given in Table 5. Model 2 demonstrates positive associations between pulse consumption and agreement that this consumption is healthy, that participants have always eaten/been brought up eating pulses and that pulse consumption is practical. Model 3 demonstrates positive associations between pulse consumption and enjoyment. The differences found between models 2 and 3 also suggest a role for enjoyment in the associations between pulse consumption, perceived practical aspects, perceived healthiness and upbringing. Model 4 demonstrates an additional positive role for cooking abilities in pulse consumption, and comparison between models 3 and 4 suggests a role for cooking abilities in the associations between pulse consumption, enjoyment and perceived healthiness. Model 5 demonstrates positive associations between pulse consumption and sensory properties. Model 6 demonstrates an additional association between pulse consumption and perceived cooking abilities. Comparison between models 5 and 6 also suggests a reduced role for upbringing, perceived practical aspects and sensory properties in pulse consumption and an increased role for perceptions of healthiness when perceived cooking abilities are also considered.

**Table 5:** Results of all regression analyses investigating pulse consumption (*n* 633)

	2 – demographic variables, plus some attitudinal factors		3 – demographic variables, some attitudinal factors plus enjoyment		4 – demographic variables, some attitudinal factors, enjoyment and cooking abilities		5 – demographic variables, some attitudinal factors, plus sensory properties		6 – demographic variables, some attitudinal factors, sensory properties and cooking abilities	
	R^2^ = 0·12, adj. R^2^ = 0·10, F(9, 632) = 9·06, *P* < 0·01		R^2^ = 0·14, adj. R^2^ = 0·13, F(10, 632) = 10·07, *P* < 0·01		R^2^ = 0·16, adj. R^2^ = 0·14, F(11, 632) = 10·68, *P* < 0·01		R^2^ = 0·12, adj. R^2^ = 0·11, F(10, 632) = 8·61, *P* < 0·01		R^2^ = 0·15, adj. R^2^ = 0·13, F(11, 632) = 9·72, *P* < 0·01	
Regression model	Beta	*P*	Beta	Beta	*P*	Beta	Beta	*P*	Beta	*P*
Gender	–0·013	0·73	–0·021	0·58	–0·021	0·57	–0·013	0·72	–0·016	0·67
Age	0·077	0·06	0·058	0·16	0·061	0·13	0·068	0·10	0·071	0·08
Eat alone/with others	–0·050	0·31	–0·056	0·24	–0·055	0·25	–0·050	0·30	–0·050	0·29
Cook for myself/for others/do not cook	0·027	0·58	0·030	0·53	0·046	0·33	0·029	0·55	0·047	0·33
Practical aspects	**0·129**	**0·01**	0·079	0·11	0·003	0·95	**0·106**	**0·03**	0·019	0·71
Healthiness	**0·129**	**< 0·01**	0·063	0·17	**0·094**	**0·04**	**0·113**	**0·01**	**0·139**	**< 0·01**
Upbringing	**0·117**	**0·01**	0·071	0·11	0·046	0·30	**0·097**	**0·03**	0·067	0·13
Social influences	0·078	0·10	0·044	0·36	0·024	0·61	0·054	0·27	0·036	0·46
Quality issues	–0·014	0·73	–0·019	0·62	–0·018	0·64	–0·010	0·81	–0·012	0·77
Enjoyment			**0·212**	**< 0·01**	**0·165**	**< 0·01**				
Sensory properties							**0·099**	**0·04**	0·049	0·32
Cooking abilities					**0·183**	**< 0·01**			**0·208**	**< 0·01**

Significant predictors (*P* < 0·05) are given in bold.

## Discussion

This study aimed to investigate the importance of a number of barriers and facilitators for legume and pulse consumption, with a specific focus on enjoyment and sensory properties, and a possible role for perceived cooking abilities in these relationships. Some interesting findings emerge.

First, without consideration of enjoyment, sensory properties or cooking abilities, legume and pulse consumption was associated with perceptions that this consumption is healthy and agreement that participants have always eaten or been brought up eating legumes and pulses. Awareness and knowledge of the health benefits of legumes and pulses are recognised as important predictors of their consumption^(20,23,24,26,28,29,40)^, resulting in repeated suggestions for increased education^(20,21,27)^, although there is recognition also that education alone is unlikely to be sufficient to result in behaviour change^(20,21,27)^. Habit, familiarity or habits from childhood are also well-known determinants of healthy food intakes, and again recognition of their importance in legume consumption^(21,23,24,26)^ has resulted in suggestions for early education to increase familiarity, alongside other strategies, such as increased exposure and food tastings^(22,24,35)^.

Second, enjoyment was a strong predictor of both legume and pulse consumption, such that relationships with perceptions of healthiness and upbringing were no longer found. Enjoyment or perceptions of liking are again well known to predict food consumption and have been reported previously both for legumes^(29,40)^, pulses^(20–22,24)^ and for vegetables more widely^(30–34)^. Comparison between the results of regression models 2 and 3 also suggests that consideration of enjoyment removes any effects due to perceived healthiness or upbringing, such that in the presence of enjoyment, healthiness and upbringing are no longer important. The greater importance of enjoyment compared with perceptions of healthiness or upbringing has previously been reported^(20,22)^; indeed, enjoyment is commonly considered the primary determinant of food consumption.

Third, cooking abilities were also a predictor of legume and pulse consumption. Various research suggests that consumers are quick to blame poor cooking knowledge and skills for a low consumption of both legumes and pulses^(20,22,24,27,29)^, and suggestions for improvements specifically in cooking abilities are frequently given by researchers^(22,24,27,35)^. Qualitative research also finds requests from consumers for increased cooking knowledge and ideas, e.g. in the form of recipes, if they are requested to increase intakes^(22,24–26)^. The value of recipe provision and cooking workshops for increasing cooking skills, knowledge and confidence is easily demonstrated^(41,42)^, and research has demonstrated the value of these activities for increasing intakes of vegetables^(43,44)^ and other healthy foods^(45–47)^. Few studies have looked at the value of these activities specifically for increasing intakes of legumes and pulses, but Hemler et al.^(40)^ in a questionnaire study reported strong correlations between legume consumption and agreement with the statement ‘I enjoy trying new recipes and dishes with legumes’.

While perceived cooking abilities have independent effects on consumption, however, comparison between models 3 and 4 suggests that consideration of perceived cooking abilities also reduced the importance of enjoyment in legume and pulse consumption, and increased the importance of perceptions that legume and pulse consumption is healthy. These findings suggest, first, that perceptions of enjoyment are less important in legume and pulse consumption when perceived cooking abilities are also considered. Thus, in the presence of perceived cooking abilities, enjoyment has less of an effect on legume/pulse consumption, and considering the beta-weights in both models 4, the independent effects on consumption of both perceived cooking abilities and enjoyment are roughly comparable. This secondary role for perceived cooking abilities is interesting. There was some suggestion also in our qualitative work that cooking abilities can mitigate concerns related to enjoyment^(24)^. Consideration of perceived cooking abilities also increased the importance of perceptions that legume and pulse consumption is healthy. These findings suggest that cooking abilities can facilitate legume/pulse consumption in the presence of perceptions of healthiness and/or that perceptions of healthiness can facilitate legume/pulse consumption in the presence of cooking abilities. The inclusion of cooking suggestions and recipes when promoting health benefits^(43–45)^ and the inclusion of nutritional education in cooking courses^(43–45)^ thus may both be of value. Consideration of enjoyment and perceived cooking abilities together also further reduced the importance of upbringing in legume and pulse consumption. While upbringing is known to be important in healthy eating^(21,23,24,26)^, these findings suggest that the combination of enjoyment and cooking abilities may play a specific role for encouraging legume and pulse consumption in those who have had less experience with them earlier in their lives. Our findings demonstrate a clear role for enjoyable cooking experiences and the creation of enjoyable dishes for increasing legume and pulse consumption. Research demonstrating direct causal benefit is required, but demonstration of the secondary roles for perceived cooking abilities are novel findings of this work. Not only are greater cooking abilities associated with greater legume/pulse consumption directly, but greater perceived cooking abilities also reduce the effects of enjoyment, enhance any effects of perceived healthiness and reduce effects due to upbringing.

Fourth, sensory properties had little impact on the consumption of legumes, but both practical aspects and sensory properties were important in the consumption of pulses. This distinction between legumes and pulses is likely a reflection of the legumes and pulses specifically queried in our questionnaire. Pulse consumption was assessed through reports on the consumption of various types of bean, chickpeas and lentils, while legume consumption was assessed through reports on these foods, plus peas and peanuts. Peas and peanuts are easily available in the UK, peas often in frozen or canned form and peanuts as pre-shelled snacks, with long use-by dates and easy storage requirements. Their taste, textural and other sensory properties are also generally well-liked. Beans, chickpeas and lentils by comparison can be reported as difficult to find, impractical or inconvenient to use and to have sensory properties that are distasteful^(21–27)^. These differences may suggest that increasing the consumption of peas and peanuts is ‘an easier sell’ in the UK, compared with increasing the consumption of pulses, while still reaping health and environmental benefits. These findings also demonstrate the importance of distinguishing between and carefully defining ‘legumes’ and ‘pulses’; confusion that has recently been suggested not only to hamper research but also public health efforts^(48,49)^.

Importantly, furthermore, consideration of perceived cooking abilities reduced the importance of both the sensory properties and perceived practical aspects of pulse consumption. Similar to the effects above in upbringing, these findings suggest a specific role for perceived cooking abilities for encouraging consumption alongside low perceptions of sensory properties and high concerns over practical aspects. These findings may suggest benefit not only from cooking abilities in the form of knowledge and skills on how to alter tastes and flavours but also in the form of simple cooking suggestions, such as the use of canned pulses or freezing techniques, to also improve practical aspects. These suggestions are made elsewhere^(21–24,26,27,33,35)^, but demonstration of potential value in a large population sample is again a novel aspect of this work. The promotion of simple cooking suggestions, furthermore, may be cheaper and more widely accessible than more intensive interventions such as cooking courses^(43–45)^, but benefits of their success for consumption are required.

The effects in pulse consumption also demonstrate clear differences between enjoyment and sensory properties as measured here, despite high correlations between these variables in our data. A strong association between enjoyment and sensory properties is often found^(23)^ or is assumed^(21,22)^, but our findings demonstrate a distinction between these constructs. These findings suggest that sensory properties do not necessarily contribute to enjoyment and/or are possibly not the only source of enjoyment when consuming pulses. Reports of enjoyment in qualitative studies have focussed not only on taste and texture but also from doing something for myself^(24)^ and from other aspects of the eating situation, such as the setting or company^(24,28,29)^.

Finally, no effects of gender, age, usual cooking responsibilities or usual eating situation were found in our analyses, and this was the case both with and without consideration of all attitudinal factors. Effects of these variables have been found before^(7,8,24,25,27,28)^. An absence of effects in our sample may suggest either that these effects are not as pronounced as has previously been suggested, that previous effects were actually a result of attitudinal differences, or the absence of effects may have stemmed from our specific sample or specific measures.

Strengths of our study include our large sample size, our varied sample in terms of legume and pulse consumption, age, usual cooking responsibilities and usual eating habits and our interest in both legumes and pulses. In relation to the sample, however, we did not gain responses from many males (only 18 % of the sample), and we do not know how many of our sample were vegetarians, vegans or from cultures where legumes and pulses may be more commonly consumed. We assessed a number of factors of potential importance in legume and pulse consumption, but other factors may also play a role. Notably, we did not include questions on gastric discomfort or distress, perceptions of identity or compatibility with the existing diet. Comments relating to gastric discomfort were not found in our previous work^(24)^. Increasing work also suggests that modern canning and preparatory activities^(2,4,25,26)^ and experience^(20,25)^ can reduce these effects, and that focus on these may cause their importance to be exaggerated^(9,50)^. Comments relating to perceptions of identity were also not found in our previous work in a UK sample^(24)^. Compatibility with the existing diet was not queried, as a factor more relevant to increasing, rather than current, consumption; however, inclusion of all of these factors in the questionnaire may have increased the variance explained or provided evidence for a lack of support. We also took no account of social desirability. Our questionnaire was anonymous, but healthy and sustainable behaviours are considered desirable, and this desirability may have affected participants responses.

In conclusion, enjoyment and cooking abilities were important for both legume and pulse consumption, while the sensory properties of these foods were more important specifically for the consumption of pulses. Perceived cooking abilities also reduced the importance of both enjoyment and sensory properties for consumption, mitigated the effects of upbringing and practical aspects and increased the value of perceived health benefits. These findings suggest that improvements in perceived cooking abilities may increase legume and pulse consumption, both directly and via a number of indirect routes associated with enjoyment, sensory properties, upbringing, practical concerns and perceptions of healthiness.

## Supporting information

Appleton supplementary material Appleton supplementary material

## Supplementary material

For supplementary material accompanying this paper visit https://doi.org/10.1017/S1368980024001058
