# Supplementary material for: The importance of enjoyment, sensory properties and perceived cooking abilities in legume and pulse consumption: a questionnaire study
Source: Public Health Nutr. 2024 May 7;27(1):e138. doi: 10.1017/S1368980024001058 (PMC11374571; doi:10.1017/S1368980024001058)
Supplement: Appleton supplementary material [file S1368980024001058sup001.docx]

**THE IMPORTANCE OF ENJOYMENT, SENSORY PROPERTIES AND PERCEIVED COOKING ABILITIES IN LEGUME AND PULSE CONSUMPTION: A QUESTIONNAIRE STUDY – SUPPLEMENTARY MATERIALS**

Table SM1: Statements per factor assessed in the questionnaire

| Barrier / Facilitator | Statements |
| --- | --- |
| Enjoyment | I like legumes/pulses  I enjoy eating legumes/pulses |
| Sensory properties | I find legumes/pulses tasty  I think legumes/pulses have a lot of flavour  I don’t eat legumes/pulses because of the texture (-)  Legumes/pulses are difficult to eat and/or digest (-)  I don’t eat legumes/pulses because of the smell (-)  I find legumes/pulses often smell unappealing (-)  I think legumes/pulses look unappealing (-)  I don’t eat legumes/pulses because of their appearance (-) |
| Cooking Abilities | Legumes/pulses are quick and easy to prepare  Legumes/pulses take effort to prepare and cook (-)  I know how to prepare legumes/pulses  I’m not sure how to prepare legumes/pulses (-)  I wouldn’t prepare legumes/pulses just for me (-)  I only eat legumes/pulses when they have been prepared or cooked for me (-)  I know some good methods and/or recipes for preparing legumes/pulses  I eat legumes/pulses only when I am cooking for or eating with other people  Legumes/pulses are handy if I want a snack  Legumes/pulses are best eaten as part of a meal (-) |
| Practical Aspects | I find it difficult to find legumes/pulses that I like or want to eat where I usually shop (-)  The range of legumes/pulses where I shop is good  I am able to afford to eat legumes/pulses  I find legumes/pulses expensive (-)  I find legumes/pulses go off quickly (-)  I often end up wasting legumes/pulses (-) |
| Healthiness | I think legumes/pulses are good for you  I think eating legumes/pulses will keep me healthy |
| Upbringing | I have always eaten legumes/pulses  I was brought up eating legumes/pulses |
| Social Influences | The people I eat with willingly eat legumes/pulses  The people I eat with do not eat legumes/pulses (-)  I eat legumes/pulses regardless of what other people tell me  I don’t eat legumes/pulses due to bad media reports (-) |
| Quality Issues | Legumes/pulses should be eaten as soon as possible after preparing them  I would only eat legumes/pulses that don’t show any signs of deterioration  Legumes/pulses to be eaten should be good quality  The quality of legumes/pulses is important to me  I would only eat legumes/pulses if I know where they have come from  I would always check on the origins of legumes/pulses before I eat them |
